# Co-designing solutions to promote awareness of cervical screening with women of low socio-economic position living in urban areas

**DOI:** 10.1186/s12889-026-27144-3

**Published:** 2026-04-17

**Authors:** Sophie Mulcahy Symmons, Amanda Drury, Andrew Darley, Thilo Kroll, Aoife De Brún

**Affiliations:** 1 UCD Centre for Interdisciplinary Research, Education, and Innovation in Health Systems (UCD IRIS), School of Nursing, Midwifery and Health Systems, University College Dublin, Dublin, Ireland; 2 School of Nursing, Midwifery and Health Systems, University College Dublin, Dublin, Ireland; 3 School of Nursing and Midwifery, Trinity College Dublin, Dublin, Ireland; 4 Trinity Centre for Practice and Healthcare Innovation, Trinity College Dublin, Dublin, Ireland; 5 School of Nursing, Psychotherapy and Community Health, Dublin City University, Dublin, Ireland

## Abstract

**Introduction:**

Women of lower socio-economic position have a higher incidence of cervical cancer and less awareness and lower uptake of cervical screening in Ireland. Co-design is an approach applied in health research that works with healthcare professionals, patients and service-users to collaboratively develop solutions to a problem they face, valuing their expertise and knowledge. This research aimed to co-design appropriate solutions to promote cervical screening among women of low socio-economic position.

**Methods:**

Women aged 25–65, living in Dublin, who self-reported regular or irregular screening attendance, without university-level education and in low-income employment or not working, were recruited via community organisations. A pragmatic theory-informed co-design approach was employed using the Double Diamond design framework and behaviour change theory. Four co-design workshops were conducted to ideate solutions to overcome the barriers to screening participation and prototype solutions to promote screening using liberating structures, techniques and participatory activities. Subsequently, focus groups and interviews were conducted with other women of low socioeconomic position, healthcare providers, policymakers and community workers to assess their perceptions and acceptability of the co-designed solutions.

**Results:**

The co-design group (*N* = 8) prioritised the following enablers and barriers to screening: visibility in the community; emotions of fear and anxiety; and support of friends and family. After ideating potential solutions, the top considerations were: supportive and accompanying friends/family; a national awareness day; education as early as possible. The co-design group prototyped two solutions: (1) a screening promotion tag placed alongside the price tag on underwear in clothes shops; and (2) a coffee morning in a local setting with a nurse to provide information on screening. Both solutions were perceived as acceptable to a range of stakeholders (*N* = 18) who suggested amendments to enhance the co-designed outputs.

**Conclusions:**

The co-designed solutions to develop campaigns to increase visibility in their community and normalise conversations about screening were acceptable. Community-led educational interventions and impactful marketing campaigns hold promise to increase awareness of screening. The study delineates genuine co-design research where women were decision-makers in developing relevant interventions to promote cervical screening in their community. This study provides a transparent, novel, theory-informed framework for co-designing health interventions.

**Supplementary Information:**

The online version contains supplementary material available at 10.1186/s12889-026-27144-3.

## Background

### Challenges for equitable cervical cancer elimination

There is a global call to action to eliminate cervical cancer and make it a rare disease [1]. Ireland is aiming to eliminate cervical cancer by 2040 [2]. Cervical screening is one tactic required to eliminate cervical cancer. In Ireland, cervical screening is freely available to anyone with a cervix aged 25–65. However, some communities are less likely to attend [3]. In Ireland, those who are living in disadvantaged areas, with low levels of education, and are on low-income employment or are not working, are less likely to attend [3–6]. Furthermore, those living in disadvantaged areas and in urban areas have a higher risk of getting cervical cancer, which may in part be due to lower screening participation [3, 7–9]. Barriers to attending screening are multi-faceted, and lack of knowledge of the service and the benefits of attending, fear of bad news, discomfort with the test, perceived lack of relevance, limited social support and screening attendance norms; enablers to attend include considering screening a positive thing to do for your health, healthcare professional incentives and reminders, and social support and encouragement from peers [10–14]. To enable an equitable path to eliminating cervical cancer, solutions to promote cervical screening should be targeted at those who encounter barriers to participation.

### Co-production and co-design as a means to develop health interventions

Co-production is a methodological approach to research that puts the service users and other stakeholders at the centre of healthcare, enabling the outcomes to be developed by them [15]. Given the move towards person-centred, value-based care and health research, co-design research is becoming popular due to the proposed benefits of more meaningful impact, and greater effectiveness, relevance and acceptability of the outcomes due to collaboration with stakeholders throughout a project [16, 17]. To ensure genuine co-production, researchers must collaborate with all stakeholders as decision-makers, rather than conducting a consultation exercise [18]. Co-design sits under the umbrella of co-production, focusing specifically on working with users (co-designers) to collaboratively develop solutions to a predefined problem, such as a service redesign or intervention [19, 20]. There are several principles in co-production, all of which should be included for genuine co-design research: maintaining relationships, including perspectives from all stakeholders, valuing all types of knowledge, shared power and ownership in the project, reciprocity, trust, and creativity [16, 18–25].

The co-design approach is inherently flexible to the needs of the co-designers through diverse use of methods but can be poorly described [26]. PRODUCES + is a framework designed to support protocol development for co-design in public health intervention research and comprises seven components: PRoblem, Objective, Design, Users, Co-designers, Evaluation and Scalability [21, 23]. To integrate more structure into co-design research, researchers may benefit from taking a design thinking lens to develop a solution to a challenge. The Double Diamond framework starts with discovering the problem, defining the problem, developing a solution and delivering through testing and refining [27, 28]. The Double Diamond reflects complex intervention development frameworks in health research as an iterative process to inform the ultimate design, which is person-centred and collaborative [29, 30]. The Double Diamond framework was adopted in this research as a creative lens to develop a solution to promote awareness of cervical screening.

Previous research to understand the barriers and enablers of cervical screening among this population has been described previously [14], and this study builds upon it to develop a solution to their challenges to engaging with screening. The aim of this study is to develop appropriate solutions to promoting cervical screening attendance among women of low socio-economic position, who have not attended university, are in low-income employment, and living in Dublin, Ireland (urban area) by using a co-design approach. There were two objectives:

- To co-design solutions to promote cervical screening in the Irish context. (phase 1)
- To explore perceptions of the acceptability of the co-designed solutions with a diverse range of stakeholders. (phase 2)

## Methods

This study is grounded in pragmatism to co-design appropriate solutions to promote cervical screening. There were two phases to the co-design process: (1) co-design workshops with the population of interest to develop solutions to promote cervical screening, and (2) feedback focus groups with a diverse range of stakeholders to gain perspectives and refine the co-designed solutions. The Guidance for Reporting Involvement of Patients and the Public version two (GRIPP2) reporting criteria were used to report public involvement in the study (Appendix 1) [31].

**Fig. 1:**
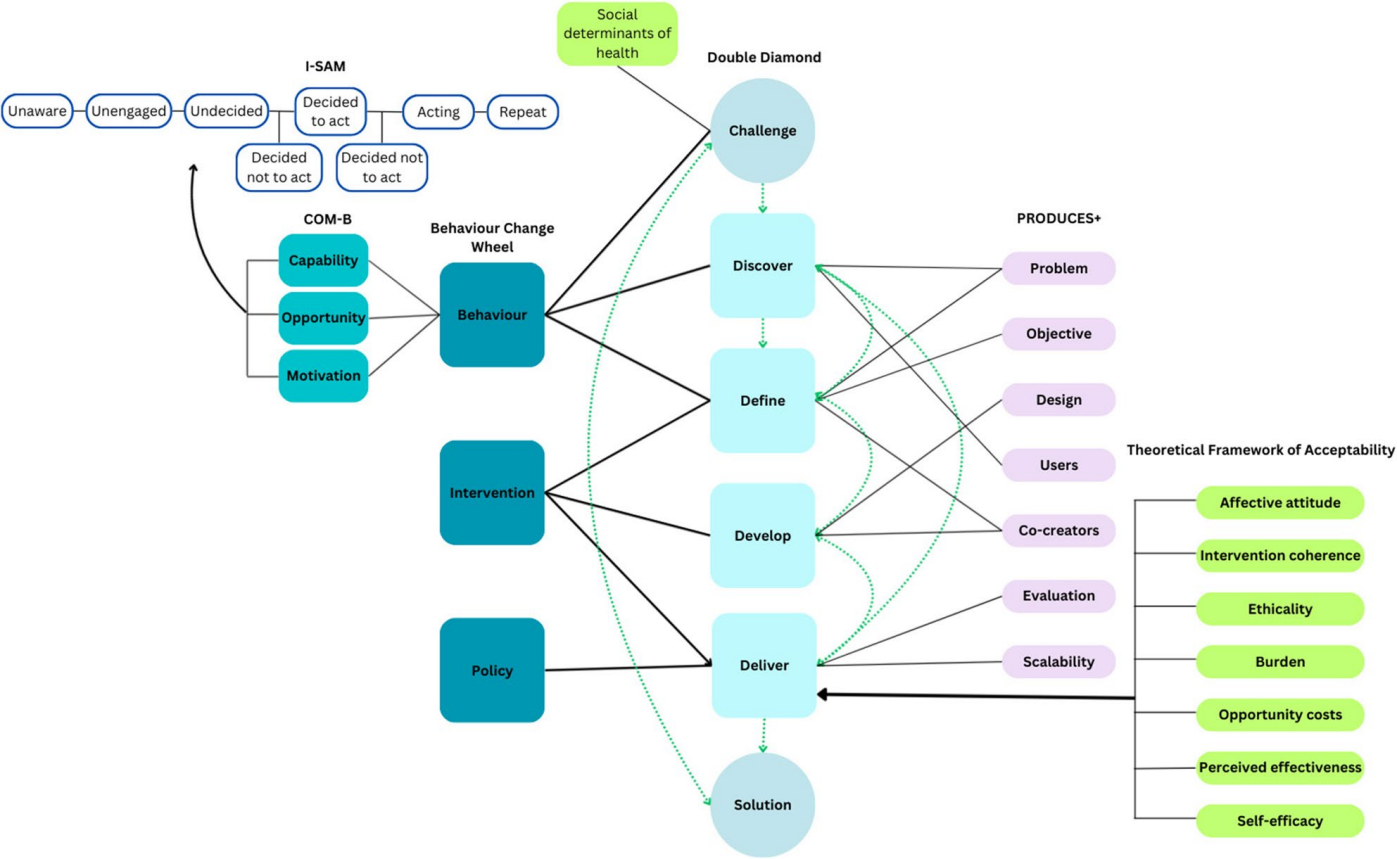
Schematic of theories and frameworks to inform co-designed development of intervention (solution)

The PRODUCES+ framework was used to support the development of the co-design protocol, see Table 1 [21, 23]. To further structure the co-design process, the co-design workshops were structured around several aspects of the Double Diamond framework (Discover, Define, Develop). To further inform the co-design process and outcomes, the Behaviour Change Wheel (BCW) and Theoretical Framework of Acceptability (TFA) were used. The BCW is a useful tool in developing interventions to change health behaviours [32]. The BCW provides a way of categorising the factors that influence the behaviour of interest (screening attendance) by capability, opportunity and motivation (COM-B) of the individual and their environment and translating solutions into intervention and policy types for implementation [32]. Barriers to screening attendance were mapped to COM-B in a previous study [14]. Solutions were matched to the BCW intervention categories: enablement, modelling, persuasion, training, coercion, education, restrictions, environmental restructuring, and incentivisation [32]. A key element of complex intervention development is assessing feasibility and acceptability [30], which remains true when using a co-design approach [21, 23]. Acceptability is a core criterion for the success of an intervention as it facilitates sense-checking and refinement of the form, content and delivery mode of the proposed intervention components [30, 33]. The Theoretical Framework of Acceptability (TFA) was applied to this study as it is designed to be suitable for both development and implementation stages of intervention studies and appropriate to all stakeholders by emphasising the need to include perspectives of the population of interest [33]. Figure 1 shows the interplay between the BCW, Double Diamond, TFA and PRODUCES+ framework in this co-design study, highlighting the iterative and evidence- and theory-based approach to intervention development.

**Table 1:** PRODUCES+ framework for co-design protocol development

Domain	Description
Problem	Women of low socio-economic position, living in urban areas are less likely to attend cervical screening and at higher risk of cervical cancer [3, 14]
Objective	Develop a solution to promote awareness of screening among women of low socio-economic position living in Dublin
Design	Four workshops to discover and define the problem, and develop and deliver solutions. Liberating structures, reflection, mind-mapping, prototyping activities used in workshops (phase 1)
Users	Women and people with a cervix aged 25–65 (eligible for cervical screening)
Co-designers	Eight women of low socio-economic position living in Dublin who were interviewed in a previous phase of the project [14]
Evaluation	Focus groups with stakeholders on perceptions of co-designed solutions (phase 2)
Scalability	Engagement with the National Screening Service & other stakeholders to share outputs and provide recommendations for service improvement based on co-designed solutions

### Phase 1: Co-design workshops

#### Recruitment of co-designers

Individuals who were interviewed in a previous study were invited to take part in the co-design study [14]. The eligibility criteria were (1) eligible for cervical screening, (2) aged 25–65, living in Dublin, Ireland, (3) in low-income employment or not working, and (4) do not have university-level education. Eleven of sixteen interviewees were interested in taking part, but six were ultimately able to take part in the workshops. Some people were unable to participate due to health or work reasons. Two members of the project advisory group who engaged as public and patient involvement (PPI) contributors also agreed to take part.

#### Ethics

The study was reviewed and approved by the institution’s Research Ethics Committee (LS-23-35-Symmons-deBrun). All individuals who expressed interest and availability to take part in the workshops were informed of the study aims and what to expect from their involvement over the phone by the lead author, prior to the first workshop. At the beginning of the first workshop, individuals were informed again about the project and asked to sign a consent form if they were happy to take part. In case of any upset caused during the workshops, a list of support resources was made available to the group. Facilitators were briefed to allow individuals to step out of the workshop for privacy and decide whether to continue. Participants were given a gratuity for their involvement in the project in accordance with the institution’s guidelines at the end of the three main workshops, and subsequently for follow-up sessions, in the form of vouchers.

#### Considerations for the workshops

Establishing culturally sensitive mechanisms to undertake co-design work and creating a welcoming and informal environment is important for upholding its principles [34]. Ní Shé and Harrison (2022) offer suggestions to enhance the quality of the co-design approach during the process, these include enabling equal involvement, managing power dynamics, choosing inclusive environments, and managing expectations. There were several mechanisms put in place to enable accessibility and decision-making, thereby sharing power among the group. Additionally, reflexivity is required in co-design studies and is particularly important when running multiple workshops due to the unpredictable nature of the process. The following sections describe the considerations taken to ensure a genuine and inclusive co-design experience.

##### Enabling equitable involvement

The study advisory group (consisting of PPI contributors, community workers and those involved in the planning and delivery of screening) were consulted when designing the protocol. Advisory group members advised on what types of activities might work to promote interaction, when and how often to have breaks, accessibility of the research materials, and the acceptability of the research approach.

Facilitator training for co-design was undertaken by the lead researcher. Many design thinking, co-design, participatory activities and facilitation techniques were explored and other co-design researchers were consulted to identify appropriate activities for the workshops to address their aims [26, 35–40]. Activities to encourage group rapport, psychological safety to speak up and collaborative discussion were identified and applied in the workshops. Given the education level of some of the co-designers, it was important to choose activities that were light on reading and writing; therefore, more dialogue and visual-based activities were chosen. If writing was required, facilitators could take responsibility for notetaking at tables. Consistent use of voting activities and reflective discussion ensured the groups’ ownership of the study and the lead researcher’s role as a facilitator to support group consensus. The co-design group were also asked how often and at what intervals they would prefer to meet (weekly, bi-weekly or monthly as choices).

##### Managing power dynamics

A discussion point among the advisory group was whether to host workshops with only the population of interest or with other stakeholders as well. It was decided to start the workshops just with members of the population of interest and ask them whether they wanted other stakeholders in attendance at future meetings. Factors contributing to this decision related to the sensitivity of the topic, particularly given the recent history with the CervicalCheck crisis (a public controversy with the national CervicalCheck screening programme whereby many women did not receive their accurate test results after a retrospective audit identified false negatives) [41], and concerns that women would not speak up, given their lower literacy levels and the hierarchical nature of the health system and the positions of power held by other stakeholders who could be invited.

PPI contributors were asked to be involved in the co-design sessions not just as participants but to support facilitation and lead activities, if they wished. This approach was taken to reduce power imbalances as the PPI contributors were seen as part of the community and the researcher was perceived as an “outsider”. The PPI contributors’ role was to consult on the workshop agenda and inform the appropriate language and activities used, to put members of the group at ease, to create an informal, friendly setting and to encourage discussion amongst the group. A facilitator role plan was developed and included their role to foster a safe and open environment for discussion, listen and communicate clearly, keep time, manage conflicts, manage participation to ensure everyone had an opportunity to share their views, and a list of support numbers (Appendix 2).

##### Choosing an inclusive environment

In consultation with the advisory group, three workshops were scheduled, as concerns were raised about the potential difficulties in sustaining involvement over a longer period. Members of the advisory group suggested using a community hall that was linked to one of the collaborator sites. This hall was located in the city centre and accessible by public transport. The community hall was familiar to several members of the co-design group and was accessible to anyone with mobility issues. As the workshops were three hours long, tea, coffee and sandwiches were provided. Multiple breaks and a soft starting time were planned to allow for rest.

##### Managing expectations

A group contract was developed via group discussion in the first workshop to enable the group to develop their own mechanisms of support and care within the group. At the beginning and end of each workshop, a recap of the aims of the research, decisions made during the workshop and plans for following work were discussed for consensus among the group.

##### Reflexivity

Reflexivity was applied throughout the co-design process to support the decisions made on the outcomes and activities based on group engagement and dynamics. Each workshop was audio-recorded. After each session, the lead researcher wrote notes and reflections about how the sessions went and their interpretation of the decisions made based on their initial reactions and after listening to the recordings. PPI contributors were also asked for their views on the workshops. These reflections informed what activities worked well and could be repeated and what needed to change if similar activities were planned for subsequent sessions. Changes to each agenda were made between each workshop. At the beginning of each session, the results from the previous workshops were summarised and discussed as a group to ensure nothing was missed or interpreted incorrectly, or if the group wanted to change anything.

#### Co-design workshops

Three workshops were conducted in July and August 2024, with a follow-up workshop in October 2024. Each workshop was three hours long. The agendas were planned to have time to bond as a group, using simple and fun activities that enabled individual and group work. The agendas for each workshop are in Appendix 3.1–3.4. Each workshop was structured around the Double Diamond framework to achieve one aim: prioritise the main issues, ideate solutions and prototype:

1. Workshop 1: Prioritise key issues for screening attendance (Discover, Define).
2. Workshop 2: Ideate solutions to promote screening (Define, Develop).
3. Workshop 3: Prototype solutions to promote screening (Develop).

##### Workshop 1 (Prioritise issues)

**Fig. 2:**
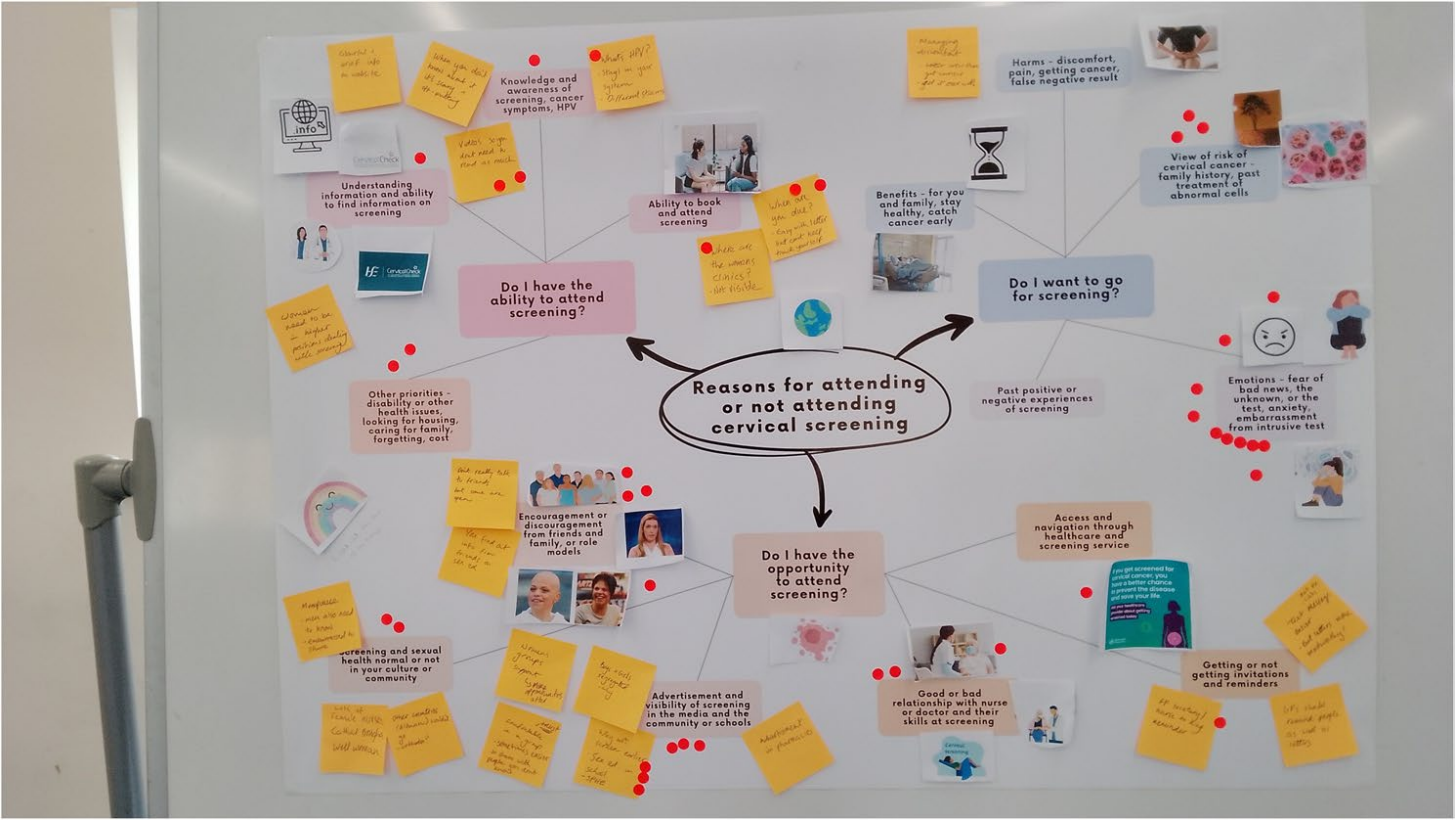
Image of mind map to prioritise main challenges with screening attendance

Workshop 1 started by taking time to get everyone familiar with each other and comfortable working as a team by chatting over tea and coffee, playing ice-breaker games, including measuring people’s energy levels at the beginning of the workshop (Appendix 3.1), and developing a group contract. During this workshop, the co-design group were presented with preliminary results from a previous study which used behaviour theory to inform the results that were displayed in a mind map written in plain English (see Fig. 2) [14]. The group were asked to reflect on the results and to discuss them as a group. Co-design members were given the opportunity to use collage or post-it notes to explain their views and stick them to the mind map. Each member of the group was then given five dot stickers and asked to vote on the issues that were most important to them. The top issues were ranked by the number of stickers placed next to them. The group discussed their initial reactions to the top issues and if they were happy with the top three issues.

##### Workshop 2 (Ideate solutions)

Before the second workshop, three questions were developed based on the top issues identified in the first workshop. These questions were phrased to find solutions to the problems, i.e. “How might we [find a solution] to [the problem]” [36, 42]. The second workshop started with measuring energy levels and a bingo session to warm up (Appendix 3.2). The 1-2-4-All liberating structure technique was used to encourage individual and group thinking to find as many solutions as possible to each question [39]. The approach gives everyone one minute to think about and come up with ideas to the question on their own, then two minutes to discuss in pairs, four minutes in groups of four, and then it is discussed as a whole group. All ideas were shared and written out on a flipchart for each question. The group then categorised similar ideas together and voted on the ideas they liked the best using the dot sticker method from workshop 1.

##### Workshop 3 (Prototype solutions)

**Fig. 3:**
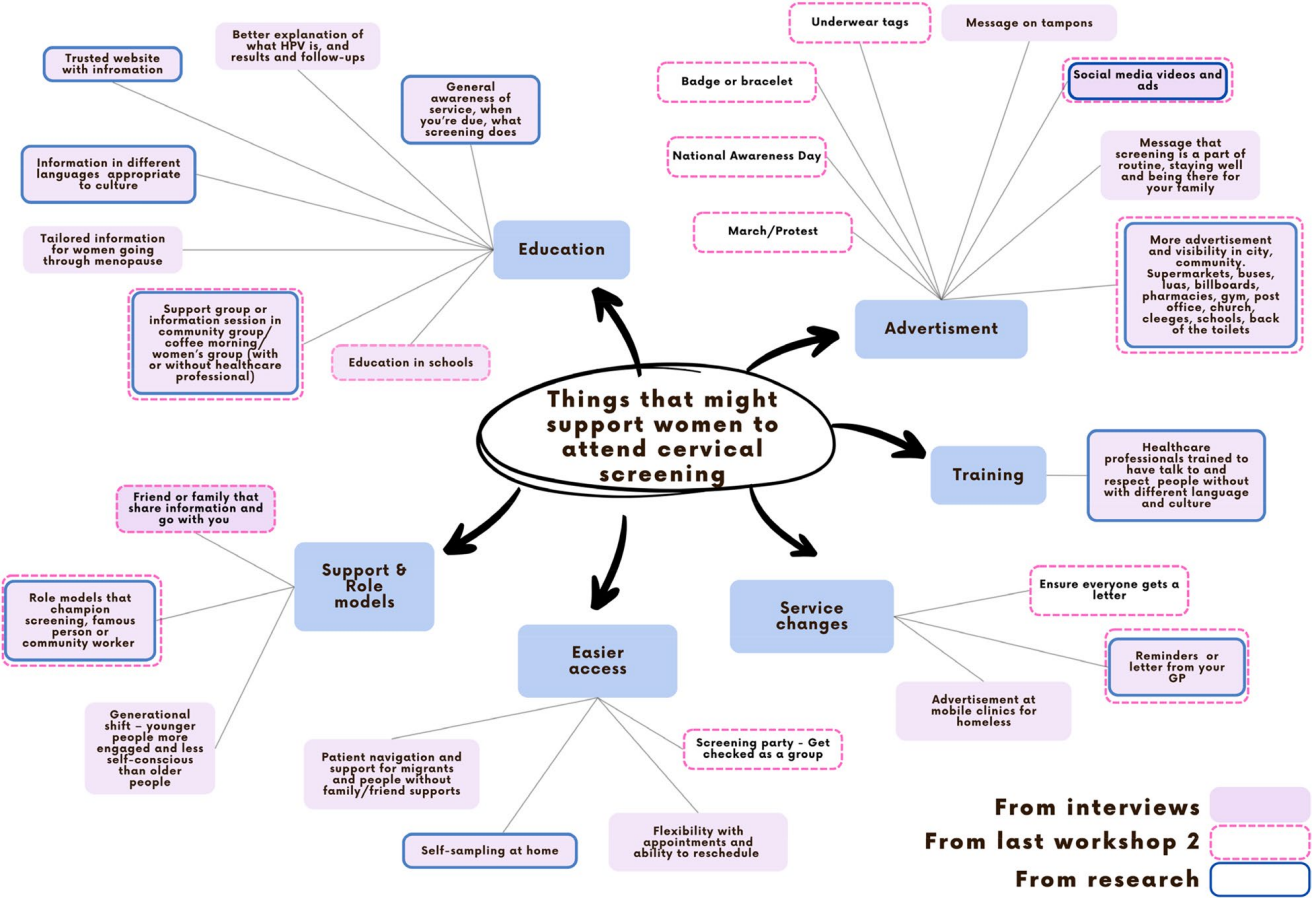
Summary of ideas to promote screening from workshops, interview study and published literature

**Fig. 4:**
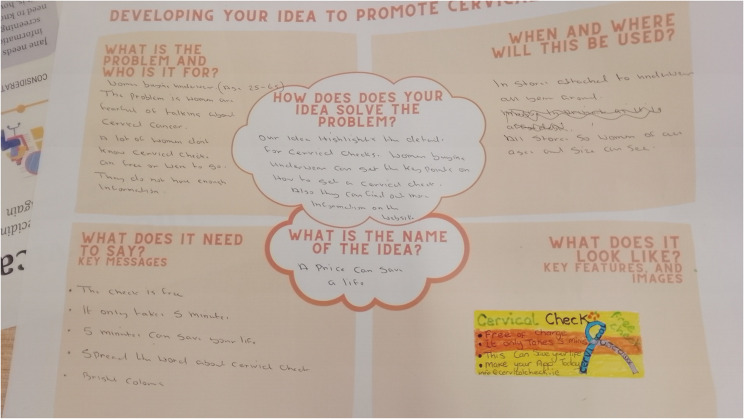
Image of worksheet completed for underwear tag solution

For the third workshop, the lead researcher summarised ideas and key messages from the workshops, interview results and published literature that promote screening in a mind map using the BCW intervention function categories (Fig. 3) [32, 43]. The lead researcher also compiled real-world examples based on ideas generated to provide inspiration to the group to develop their own prototypes (Appendix 4 for sample). The group was split into two smaller groups to design one solution each. Each group was given worksheets with prompts to develop their solution, which included the specific problem they were addressing, how their solution would solve it, what it would look like or need to say, and where and when it would be used (Fig. 4). After they developed their solution, they pitched their solution to the other group, who asked questions about the solution. Finally, individual and group reflection on their solutions was conducted using the What, So What, Now What technique (Appendix 5) [39].

##### Workshop 4 (additional workshop: refining the prototypes)

The lead researcher summarised the two solutions developed in workshop 3 into a design brief for a designer to visualise the co-designed solutions. With the agreement of the co-design group, another workshop was held to share and refine the mock-ups. The designer joined the session to receive direct feedback from the group. The workshop started with introductions, an ice-breaker session and a recap on the work completed to date. Each prototype design was shared, and the group discussed what they liked and disliked about the prototypes to further refine the solutions. The What, So what, Now what technique was used to prompt the group discussion (Appendix 6). The lead researcher made a summary of the main suggestions based on the reflections and the designer revised the designs.

### Phase 2: Focus groups to assess acceptability of the co-designed solutions

This qualitative descriptive study assessed the acceptability of two co-designed solutions to promote awareness of cervical screening with a range of stakeholders via focus groups and interviews.

#### Sample

Stakeholders represented in the focus groups and interviews consisted of women in the population of interest, community development workers, representatives from cancer charities, and those involved in the planning and delivery of cervical screening (including healthcare professionals who work in the area of interest, Dublin, with underserved communities, who were not involved in phase 1. Stakeholders were recruited via convenience sampling through the lead researcher’s and the advisory group’s existing networks. Invitations to participate were also shared on social media. Potential participants were screened for eligibility, and to ensure a diverse range of expertise was achieved in the sample. Participants were given the information about the project and asked to fill out an online consent form if they were willing to participate.

#### Data collection

Focus group dates were organised throughout November and December 2024. For participants who could not attend the focus groups, one-to-one interviews were arranged. A focus group with participants representing the population of interest was arranged by a collaborating organisation (further education centre) in person; all others were online via Zoom. The lead researcher conducted all focus groups and PPI contributors were invited to support the focus groups by introducing the solutions. Each solution was described under the headings What, Where, When, and Who with designs of the proposed solutions.

The TFA was used to develop the topic guide questions under its seven component constructs: affective attitude, burden, ethicality, intervention coherence, opportunity costs, perceived effectiveness, and self-efficacy (Appendix 7) [33, 44]. Focus groups lasted 63–89 min and interviews lasted 45–60 min. The online focus groups and interviews were recorded and auto-transcribed using Zoom and verified by the lead researcher. The third-party company transcribed the in-person focus group.

#### Analysis

Data were analysed using a deductive approach to framework analysis [45]. Initial open coding was conducted on all transcripts. These codes were mapped to each TFA construct and used to expand their definitions in relation to the data on each solution separately to develop an initial coding framework. A second researcher (ADB) openly coded one transcript independently to support the development of the coding framework. The research team discussed the codes and their fit with each TFA construct to refine the frameworks for each solution. Summaries of all codes and constructs were developed in relation to the research question. The data was charted and framework matrices were exported to summarise each code for each unit of analysis (focus group/interview). Responses from the population of interest (PoI) focus group were compared to the other stakeholders, representing policy, practice, advocacy and community development (PPAC), to determine similarities and differences in the acceptability of the co-designed solutions.

## Results

### Participant characteristics

There were eight co-designers in total; attendance numbers varied across the four workshops (Table 2). Two women from the co-design group reported they were underscreened, two were of non-Irish ethnicity^1^, and ages ranged from 31 to 58. The co-designers wanted the solutions to be designed by them, rather than outside influence; therefore, no other stakeholders were invited to subsequent workshops.

**Table 2:** Attendance at workshops and co-design group member characteristics

Workshop attendance	Number
Workshop 1	8
Workshop 2	5
Workshop 3	8
Workshop 4	4
Individual Characteristics	
Screening history	
Regularly screened	6
Underscreened	2
Age	(31-58)
30-39	2
40-49	3
50-65	3
Ethnicity	
Irish	6
Non-Irish	2

Four focus groups and two interviews were conducted in phase 2, consisting of 18 individuals, to obtain feedback on the two co-designed solutions. Six participants represented the population of interest (PoI) (those of low socio-economic position who were living in Dublin and were eligible for cervical screening). In contrast, 12 represented policy, practice, advocacy and community development (PPAC). Table 3 displays the demographics of the sample. One focus group was conducted in person with the PoI sample and the other focus groups were held online with a mix of PPAC participants in each. Two interviews were conducted with PPAC stakeholders.

**Table 3:** Demographics of focus groups and interview participants

Demographic characteristics	Number (N=18)
Women representing population of Interest (PoI)	6
Policy, practice, advocacy and community development stakeholders (PPAC)	12
Patient advocacy/charity/community development	4
Healthcare professional (HCP)	4
Policy/Research/Implementation of screening	4

### Co-design workshops

In the first workshop, the group decided that meeting every two weeks was optimal, and the first three workshops were organised in this way. The group developed a group contract, consisting of principles to respect everyone’s opinion, allow everyone to speak, confidentiality, create a safe place to share experiences, get to know each other, and have fun. After voting on the reasons that encouraged or deterred screening were, the top reasons were: Visibility and advertisement in the community (6 votes), Emotions of fear, anxiety and embarrassment (10 votes), and Support of friends and family (4 votes), knowledge and awareness of screening (4 votes) (Fig. 2). The group were surprised that emotions were voted as the top reason. The group felt that knowledge and education mediated emotions, and knowledge should be given greater importance. The group decided that education and visibility of screening should be combined as joint first priorities. These top priorities were translated into “how might we” questions (Table 4) to ideate solutions for workshop two.

**Table 4:** “How might we” questions developed regarding each priority

Priority	“How might we” Question
Visibility in the community	How might we increase awareness of the cervical screening service in a way that grabs your attention in your area?
Education	How might we provide women with better information on cervical screening and HPV, so women understand what screening does?
Emotions	If someone was afraid of screening and getting bad news, how might we reassure and encourage them to go?
Support of friends and family	If you had friends or family that didn’t go for screening, how might we support them to attend screening? (Not covered in workshop, ran out of time, and co-design group felt there was overlap with the Emotions question)

All ideas were grouped together into similar categories. Ideas were grouped into information and messages that should be communicated about screening and actions to promote screening. Key messages the group thought should be explained about screening related to information about the test itself, how it was done and what HPV is, that screening is free, that it is a preventive check. Other messages were motivational, such as describing screening as a positive thing one can do for their health, that it is easy and shouldn’t be painful, and that it could save your life. The group also discussed how to share these messages to be accessible and acceptable, which included having women explain the information, hearing about others’ experiences, using simple language and bright colours. The ideas to promote screening included advertisements in public spaces, making badges or other products for cervical screening promotion, education in schools, discussing screening in women’s groups, healthcare professionals providing information sessions, peer support and encouragement, automated text message reminders, videos with information about screening, social media videos and advertisements, national awareness days, a march about screening, ads on underwear, and getting screened as a group. The top solutions to promote screening that the group voted for were, support of friends and family to talk to and go with you, a national awareness day, and education as early or as young as possible to normalise screening. All of the ideas were grouped using the BCW intervention functions, potential solutions were added that were mentioned in previous interviews (Fig. 3) [14], and others that were identified in existing literature to support the co-design group to prototype solutions in workshop 3.

In workshop 3, the group developed two solutions to promote screening. The first was a promotional tag about screening to attach to the price tags on underwear in clothes shops. The second solution was an informal coffee morning in a local community space to address concerns about screening by a local nurse or trained community champion. A fourth workshop was arranged to refine the co-designed solutions after mock-up designs were made.

### Promotional tag on underwear

**Fig. 5:**
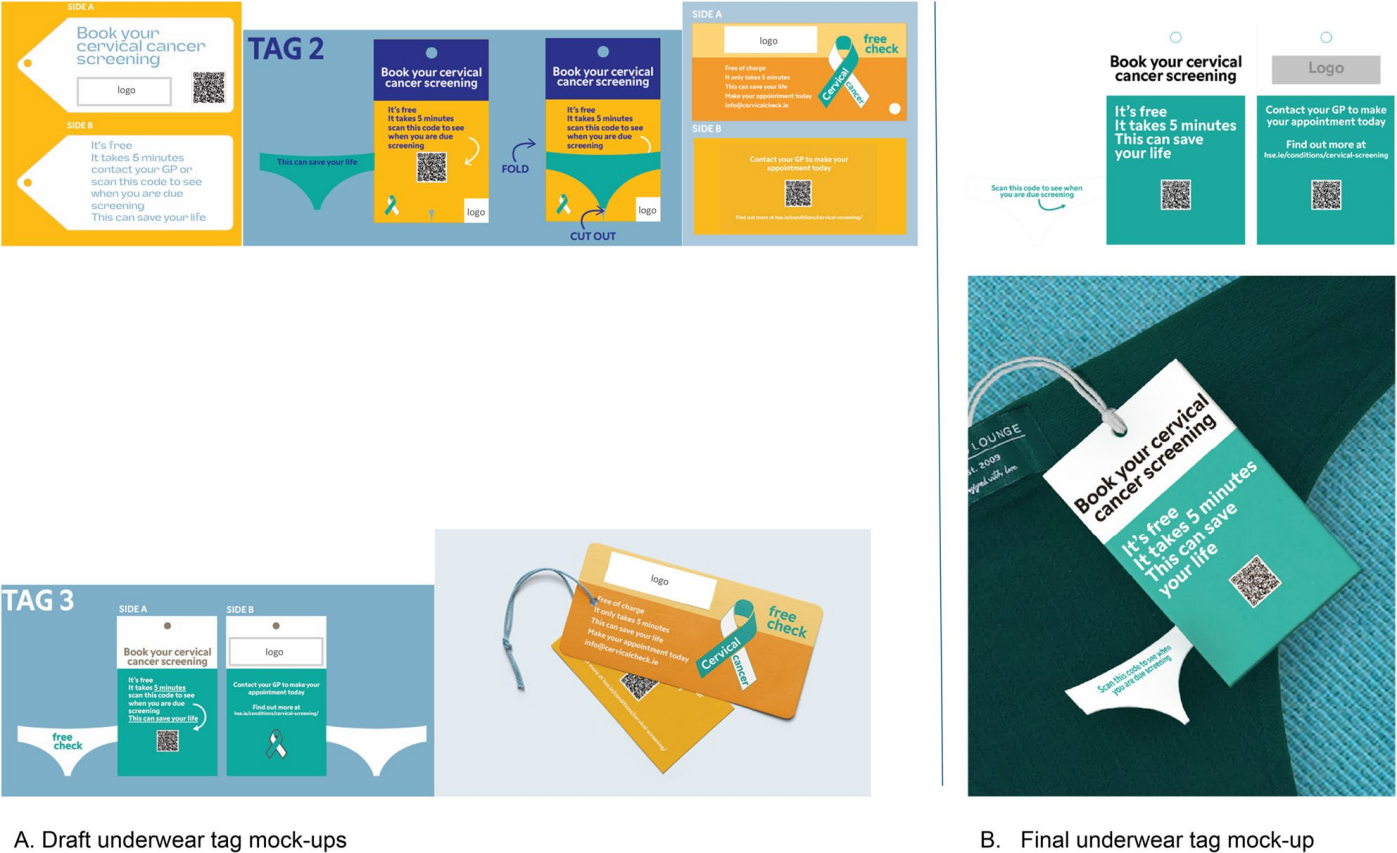
Underwear tag mock up designs

The first sub-group was inspired by the previous idea of a promotional tag from workshop 2 and wanted to develop the concept further. They decided to create a tag that attaches to the price tag on underwear in clothes shops to promote awareness of cervical screening (Fig. 4). The aim was that the campaign would have a broad reach, including those in their community, to encourage and normalise public dialogue on screening. The tag was designed to be simple, use plain English, bright and eye-catching. The key content for the tag reflected the main questions the co-designers had about screening: screening was ‘free’, that it was easy to do, and that it could ‘save your life’. A QR code link to the online screening register and contact details to find out about screening via the health service’s website, or to contact a local GP, were proposed to access further information. The co-designers decided the campaign should run for a specified length of time, i.e., a month and coincide with a national awareness day for cervical cancer. The group felt it was important that the campaign was linked to a reputable organisation, such as a cancer charity, the Irish health service or CervicalCheck. The original tag design can be seen in Fig. 4, the mock-up designs made by a designer are shown in Fig. 5A.

After the mock-up prototypes were shown to the co-designers, several refinements were made to the final design of the solution, Fig. 5B. Changes to the underwear tag solution included a preference for the teal and white colours, decisions that a weblink and QR code would go to the website to register for screening and increasing the font size of the “it’s free” message. The teal ribbon for cervical cancer was unfamiliar and, therefore, removed.

Overall, phase 2 participants had positive views towards the underwear tag solution and thought it was innovative and eye-catching, but may exclude some underserved communities. In their view, the tag needed to ensure simple imagery and text that was instantly recognisable as cervical screening to facilitate engagement. Participants thought the check the register message was the key message and other information needed to be reviewed to ensure accuracy of the content on the tag. Collaboration was viewed as a crucial facilitator to ensure buy-in and capacity to implement the campaign. The main findings with exemplary quotes are in Table 5.

**Table 5:** Promotional underwear tag code descriptions and example quotes

TFA construct	Codes	Key findings	Example quote
Affective attitude	- Overall impression that campaign is innovative - Perceptions of need for increased education and awareness in community	Participants felt the tag campaign had potential to be impactful as it was innovative. Participants held view that screening needed to be normalised among the public and awareness campaigns could support this.	“*Yeah*,* I think it’s really it’s something I would never have thought of actually*,* it’s a really innovative idea. And I think I suppose it’ll reach*,* I suppose*,* if you look at the odd*,* the odds of reaching somebody*,* it’ll reach somebody at whatever level they’re able to reach it at. So I think it’s definitely a good idea. Absolutely*.” FG2P3, PPAC participant (HCP)
Burden	- Ease of interpreting that tag is about screening - A free service - Cost of producing tags	The tag was perceived as simple and clear but some thought there could be less information to ensure it was understandable at a glance and reduce the risk of it being off-putting if it had too much content. Participants felt it essential to emphasise that screening is a free service. Some participants thought the tag production could be costly or wasteful.	“FG1P2: *I think it’s very important to say that it’s free*,* because a lot of people would worry about the cost of it*,* that’s one thing that has to be highlighted is that*. FG1P1: *I’d say keep it very plain and simple. Not a big load of information.* FG1P6: *You don’t want information overload you’d lose people*.” PoI participants
Ethicality	- Avoiding potentially triggering messages - Inclusive ethos - Empowering women’s health promotion	All participants expressed a view that the tag should be inclusive of everyone’s needs and abilities, this included having information available in different languages. Participants agreed that the tag could empower and motivate health promotion. Some PPAC participants raised concerns about the tag information being reductive, by implying the screening test was easy when some find it difficult, upsetting those with history of cancer or sexualising screening due to the association with underwear.	*“I think a lot of campaigns before would say it takes 5 minutes*,* and it’s easy when we know it’s not easy for a lot of women.”* FG3P4, PPAC participant (research, policy and implementation)
Intervention coherence	- Visuals on tag should portray cervical screening - Tag addresses key information - Identifying key message - Campaign in alignment with national cervical cancer awareness days	Participants agreed that the content on the tag captured the most important information about screening and the main message that screening was free and to check the register. PPAC participants suggested changes to improve the accuracy of information on the tag, such as, adding the eligibility age range, removing the word ‘cancer’, and change ‘book screening’ to ‘check if you are due screening’. There were mixed views on stating to contact ‘your GP’ for information as some viewed GPs as trusted while others had concerns that the GP was not necessarily the sample-taker. Some felt the underwear was a good symbol for screening, particularly if accompanied by the CervicalCheck logo, but some PPAC participants felt a more explicit visual representation of cervical screening was needed.	“*You know*,* saving your life is pretty powerful*,* you know. It’s doesn’t get any greater than that. So for me*,* if I think that’s probably the most powerful thing in that that statement*,* you know*,* in that block of text to me. The free is fine*,* it takes 5 minutes*,* that’s all great building up*,* but it can save your life is*,* yeah*,* it’s really powerful*” FG2P1, PPAC participant (research, policy and implementation)
Opportunity cost	- Trust in organisations associated with campaign - Retailer backing - Collaboration with implementers to ensure feasibility and capacity	Participants felt it was important to work with trusted organisations to legitimise the information of the tag. PPAC participants thought collaboration with implementers would support feasibility by ensuring capacity for increased interest in screening. The retailer’s motivations were queried and participants wanted them to have values in health promotion.	“*Anything that’s campaign based has a significant impact on the programme. So my ask would be that nobody does anything in isolation we’ve got to do it collectively*,* and by that I mean in partnership*,* be it academia*,* or with the likes of Irish Cancer society*,* Mary Keating [cancer charities in Ireland]. The strength in all of this is that we go out together because that that’s the very*,* very important bit.”* FG3P3, PPAC participant (research, policy and implementation)
Perceived effectiveness	- Targeting general population vs. underscreened groups - Need for evidence to ensure effectiveness - Views on whether it will work to promote cervical screening - Ways to increase effectiveness	The tag was viewed as eye-catching and would stand out from retailer tags and raise awareness of screening among the general population by most participants, while others thought it might be ignored. Some felt the tag may not be accessible to underscreened communities and emphasised the needed for tailored supports. PPAC participants wanted to see evidence of whether these types of campaigns were effective. Suggestions to increase effectiveness of the campaign included using posters throughout the shop and in the changing rooms, using stickers and tags, and having an online presence and pairing the campaign with mass media.	“*Probably when you are shopping you probably don’t pay too much notice of the tag. But when you go home (FG1P2: when you go home you would be) the fact you have to open it to take it off makes you stop and look at it.”* FG1P1, PoI participant
Self-efficacy	- Literacy barriers to engage with tag - Barriers to seeing tag and booking screening	The PoI group seemed confident using the QR code, however PPAC participants had concerns about digital literacy and the public’s ability to engage with the tag and suggested a phone number to increase accessibility. Other potential barriers related to one’s perception that screening was relevant to them and their ability to follow up on the information and book screening with any registered sample-taker.	“*Women who don’t have a GP*,* that will be a barrier for them*,* and women who can’t get a GP*,* again that’ll be a barrier for them*,* and women whose GP is a man and don’t want to go*,* that’ll be a barrier for them. So we need to probably be more specific about how you book your appointment and it isn’t through contacting your GP*,* it’s about go online to find a sample-taker in your area… you don’t need a GP to make an appointment.*” FG4P1, PPAC participant (research, policy and implementation)

The overarching recommendations to improve the underwear tag solution were to increase clarity of the message and ensuring pathways for individuals to follow up with screening. To enhance clarity of the solutions there is a need to develop or get consensus on a visual symbol that represents screening or could be associated with screening, emphasise the key message of the tag that it’s a free service and to check register, highlight that screening can be done with any sample-taker, and use more concise language and less words for more impactful and understandable. Participants also wanted to ensure there are multiple ways for follow-up i.e. a phone number and QR code, as well as suggesting that the campaign should include posters in changing rooms.

### Coffee morning

**Fig. 6:**
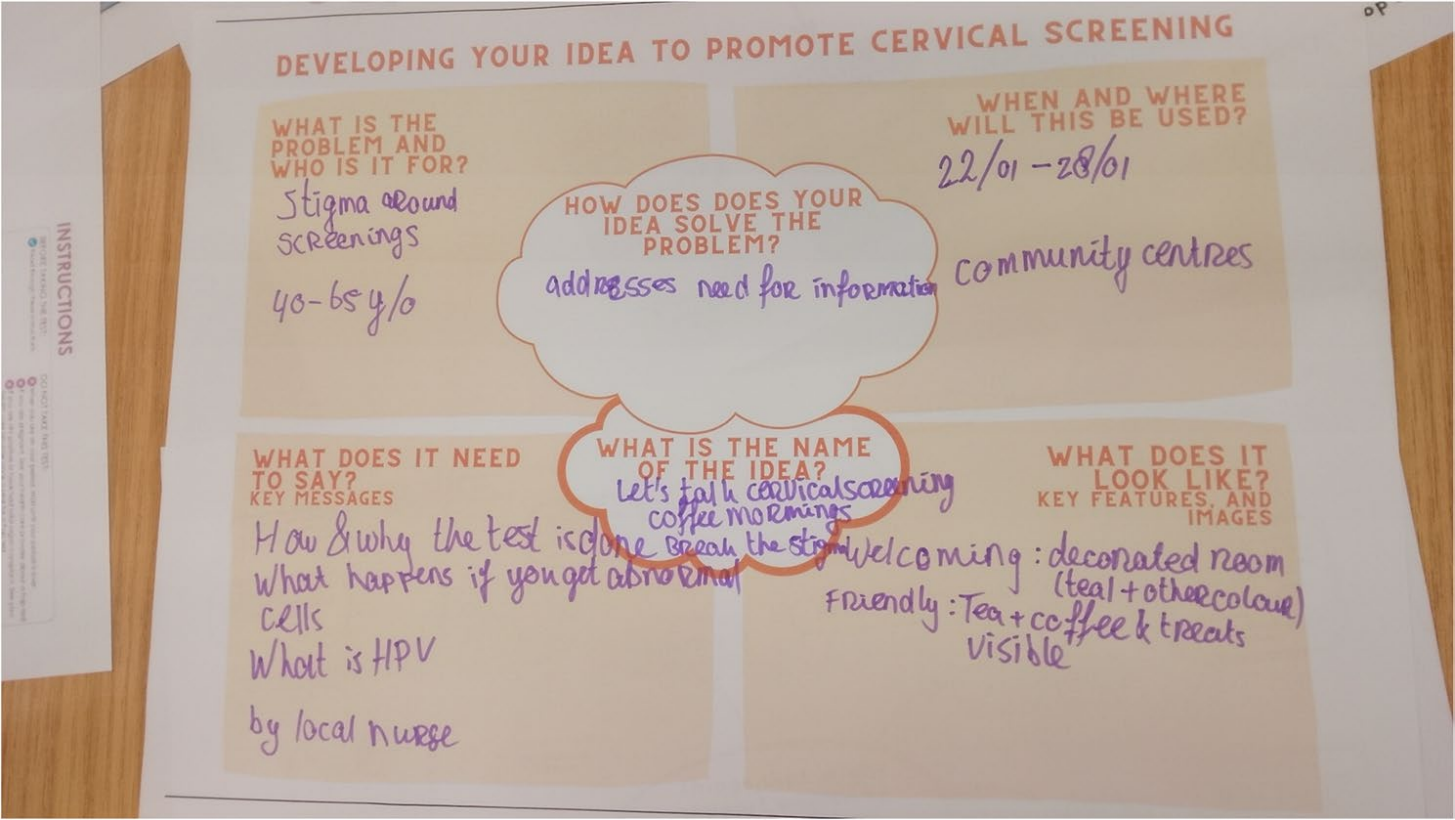
Image of worksheet completed for coffee morning solution

**Fig. 7:**
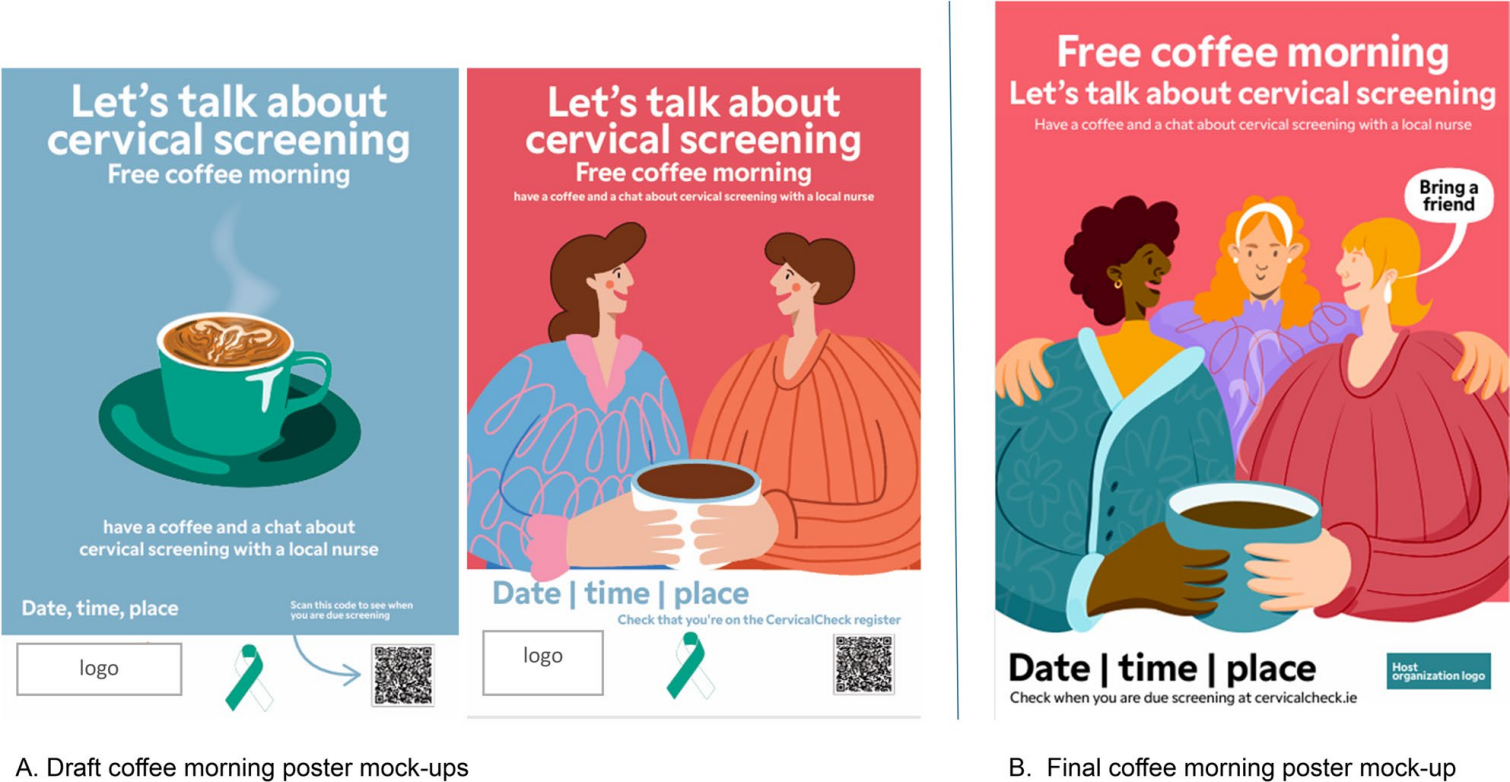
Coffee morning poster mock-up designs

The second sub-group was interested in developing a solution to appeal to those who might be afraid of screening or have concerns they would like addressed before making the decision to attend screening and address sustained stigma of screening (Fig. 6). They decided to develop an informal event, a coffee morning, to provide information about screening and create a dialogue with accurate information and peer learning in a welcoming space in the community. Creating a safe environment was core to this solution. A community centre that is comfortable and familiar to those living locally was the optimal location for the event. The person delivering the information at the event should be known and trusted in the community, such as a local practice nurse or a trained community champion. Co-designers also felt that the person delivering the information should understand the community, be able to speak their language, be knowledgeable about cervical screening and be able to answer questions about screening. The coffee morning was planned as a one-hour session, the first half consisting of provision of key information about screening and a Q&A, followed by an informal space for attendees to talk and share information among themselves and ask the facilitator private questions. Key topics to be addressed at the coffee morning were screening is a free service, how to register and book, how the screening test is performed, what HPV is, and what happens if you get positive results (i.e., messages outlined from workshop 2, see Results section, Co-design workshops). The event was viewed as something that could recur throughout the year, but with emphasis during a national awareness day about cervical cancer. The group also developed a design for a poster to advertise the event. The mock-up posters designed by a designer are presented in Fig. 7A.

After sharing the coffee morning poster designs and reflecting on the solution further, several changes were made. The co-designers thought the event should start with having time for attendees to settle in and get tea. It was also suggested that the equipment used for screening should be brought to the event so attendees can see them. The poster with several women around a coffee cup was decided to be more suited to the event as it portrayed a sense of welcoming, as they did not find the image of the reproductive system understandable or inviting (Fig. 7B). To add to the welcoming feeling and recognising the priority for peer support, the “bring a friend” message was added. QR codes were only viewed as legitimate if it was accompanied by the logo and affiliation of a trusted organisation, hence raising the importance of a collaborative event.

Phase 2 participants had positive views towards the coffee morning solution to promote dialogue on screening in the community with minimal costs. To make it easy to engage with the coffee morning, participants discussed how it could be brought to existing community groups. Participants agreed that the format of the event, location and facilitator were important to creating a welcoming environment to address concerns and ensure it was inclusive. PPAC participants believed there was existing capacity and interest to deliver this kind of event. To increase effectiveness of the event, some suggested an online component to the event and having a clear pathway to support attendees to register or book screening. The results and illustrative quotes are displayed in Table 6.

**Table 6:** Coffee morning code descriptions and example quotes

TFA construct	Codes	Key findings	Example quote
Affective attitude	- Overall impression of the coffee morning and poster - Need for education and awareness to improve limited knowledge and stigma in the community	Positive sentiment towards the coffee morning, particularly as there was a view that more awareness of the service was needed in the community to encourage screening as a norm.	“*I think anything at all that may trigger a woman to even think about “oh*,* yeah*,* I’m due a smear test”*,* I think it’s a great idea overall and the fact that it’s not too formal*,* and that*,* you know*,* women have asked for it to be in a relaxed sort of atmosphere where they can bring a friend. I think all that is really positive*.” Int1, PPAC participant (HCP)
Burden	- Use accessible language - Meet women where they go so it is easy to engage - Cost of delivering coffee morning - Screening and the coffee morning are free	Participants perceived minimal burden to organising a coffee morning as it was viewed as a low-cost intervention. Some felt it was important to be clear that there were no costs to attendees. To reduce potential burden of attending the coffee morning, participants suggested bringing the coffee morning solution to existing women’s groups.	“*That’s what I’m saying she has her girly group. They are all local girls*,* and cut out all the footwork and the posters and all that*,* you have a readymade group if you wanted to put something on.”* FG1P2, PoI participant
Ethicality	- Space for privacy - Inclusive ethos - Empowering women’s health promotion	Participants felt that increasing education and awareness supported informed decision making and empowerment to care for one’s health. Having the time for one-to-one questions with the community champion was deemed essential to address concerns sensitively. Participants had an ethos of inclusivity which led them to suggest the use of more inclusive language (e.g. LGBTQI+ friendly and considering the language of the attendees) to the coffee morning solution.	“*Yeah*,* like empowering yourself a little bit or something*,* taking charge of your health.”* FG1P3, PoI participant
Intervention coherence	- Creating space for informal dialogue to address concerns together - A safe and familiar location - Information in Q&A addresses key aspects of screening - Poster should be clear it is about screening	Participants agreed with the content and format of the coffee morning. Participants agreed the coffee morning would create an informal and welcoming space to foster a sense of mutual support from peers. Participants agreed it was important to provide information on HPV, what to expect during the test, the potential results and follow-up, the benefits and harms of screening, and practical information and support to book and attend screening.	“*I think you need to look after people when you come and gather them together. So it’s important to reward them for showing up so. And a venue is really important as well*,* and sometimes removing it from a clinical space can be very nice*,* and community centres as brilliant as a role and a function that they have*,* they’re never particularly warm. And what you want to convey*,* I imagine*,* with screening is that it is welcoming*,* it is where somebody that you know*,* this is a good space for you to be*,* and I think that’s important*,* the trimmings of these coffee mornings*” FG2P1, PPAC participant (research, policy and implementation)
Opportunity cost	- Existing capacity and resources support coffee morning - Collaboration with organisations and service providers to ensure feasibility and capacity	PPAC participants felt they had the resources and capacity to deliver the coffee morning solution. Participants agreed that the information was best delivered by community champions or local nurses who were familiar with the community and that local buy-in was essential to the success of the coffee morning.	“*I think it’s best to come from a group on the ground that know these kind of the women where possible*,* or the you know the community. I don’t know if they see I mean the NSS*,* the screening service*,* if that would work well with them.”* FG3P4, PPAC participant (research, policy and implementation)
Perceived effectiveness	- Need for evidence to ensure effectiveness - Target audience needs to be those who are underscreened - Views on whether it will work or not - Ways to increase effectiveness and engagement	Most participants felt it was worthwhile running a coffee morning as it was tailorable to the community’s needs and may increase knowledge of the service. Some felt it encouraged those who already have an interest in attending screening. Some PoI participants felt the event wouldn’t be engaging for them unless it addressed broader women’s health issues as they attended screening, but liked the ‘bring a friend’ message. Some felt that the effectiveness could be increased if there were service navigation support to register and book screening at the event.	*“I think it’s worth a try. I think. I think this will probably help the women who are probably gonna get it done*,* anyway*,* maybe but have a few more questions. So that’s great. Definitely. I suppose. Obviously*,* there’s going to be the more difficult to reach that this probably won’t impact*,* but you know it*,* it seems like a fairly straightforward way to try and capture some women hopefully”* FG3P2, PPAC participant (HCP)
Self-efficacy	- Literacy barriers to engage with coffee morning - Acknowledging barriers and promoting relevance of screening - Appropriate person to deliver event	Participants discussed the self-efficacy of the potential attendee and the facilitator. The ability for the potential attendee to go to the coffee morning and engage with it depended on their competing priorities and literacy level. Participants felt the facilitator needed to have the skills to deliver information about screening in a culturally sensitive way and speak the language of the community.	“FG1P6: *Me personally someone that talks our language*,* so don’t give all the technical jargon.* (talking over each other, agreement) FG1P1: *Yeah*,* the likes of [local nurse] she does come in and she’s warm and she says what she has to say*,* just you know*,* the likes of her. She can just say it*,* yeah. She’s very good you know.*” PoI participants

Suggestions to improve the coffee morning were to develop the coffee morning as a template to bring to existing groups that is flexible to be tailored to the specific needs of the group (based on input from the community) and uses “their language”. Participants wanted a poster advertisement that better represents cervical screening and the format of the day which can be advertised online and physically/by word of mouth. Some recommended expanding topics of discussion to be more holistically about cervical cancer health (i.e., HPV vaccinations) or generally about women’s health. It was essential for participants that such a solution had a clear integrated pathway for attendees to follow-up, book or participate in screening.

### Relationship to the Behaviour Change Wheel

The co-designed solutions were compared to the BCW and are summarised in Table 7. The Promotional tag on underwear solution addresses physical opportunity and aligns with the enablement and environmental restructuring intervention functions. The coffee morning solution addresses social and physical opportunity and psychological capability and aligns with the education, enablement, training and environmental restructuring intervention functions.

**Table 7:** Co-designed solutions and their relationship to BCW [32]

Solution	Intervention targets (COM-B)	Intervention function	Policy
Underwear tag	Social and Physical Opportunity	Enablement, Environmental restructuring	Communication/ marketing
Coffee Morning	Social and Physical Opportunity, Psychological Capability	Education, Enablement, Training, Environmental restructuring	Communication/ marketing, Service provision, Environmental/ social planning

## Discussion

This study developed acceptable, novel, and theory-informed co-designed solutions to promote cervical screening among women of low socio-economic position living in the inner city of Dublin. The rigorous and transparent methods reported here contribute to the wider co-design methodology literature, enabling adaptation to other health intervention studies. Fear of cancer, limited social support, lack of awareness and understanding of the relevance of screening and how to book screening are common barriers to engaging in cervical screening and the co-design group signalled these as core priorities [10–12, 14, 46]. Throughout this study, one of the key challenges continually cited by co-designers was social opportunity and the need for greater awareness and open dialogue about screening in the community. This highlights a potential communication barrier: information about screening is not reaching women effectively [14, 47]. Participants advocated for ways to promote conversations about screening and reduce perceived stigma in their community, as they felt normalising screening and education was a key step to deciding to attend. Creating a community discourse on screening was viewed as a positive action for enablement of screening participation and promotion of screening in the community could be achieved through multiple means and advertisement, both physically and online (i.e., the underwear tag solution), and through creating psychologically safe spaces to address concerns about screening (coffee morning solution). The underwear tag provides a simple, eye-catching prompt to raise awareness of the free service and a reminder for individuals to check if they are due for screening or register online that has broad reach but is in line with the level and needs of those of low socio-economic position. The promotional tag aligns with the BCW enablement intervention function and communication and marketing policy category. The coffee morning solution aims to address concerns about screening in a peer-supported and informal community setting, addressing key priorities of education, visibility of screening services and advertisement in the community, and support from friends and family. The coffee morning solution fits with the education, enablement, training and environmental restructuring intervention functions as well as the service provision and communication and marketing policy categories. Both solutions generated positive sentiment with the potential to empower and enable women to attend screening. Phase 2 participants stressed the need for collaboration to increase effectiveness and the use of simple language to increase accessibility and self-efficacy to engage with the solutions. These co-designed solutions provide recommendations to promote screening among women of low socio-economic position, by women of low socio-economic position. Due to the acceptability of the co-designed solutions, they can be used as template for further refinement or adaptation to promote screening awareness in the community. This research approach is applicable to solving other health challenges.

### Community engagement

Participants advocated for ways to promote conversations about screening and reduce perceived stigma in their community, as they felt normalising it and educating people was a key step to making a decision to attend. Social opportunity via peer support and social networks are important in influencing decisions to attend screening [11, 14, 46]. The participants from both phases emphasised the need to have in-person spaces to discuss health issues. Disadvantaged areas face health challenges with their physical opportunity due to long waiting times for primary care services, fragmented services and limited safe social spaces [48]. To address these needs and reduce health inequalities, community voices should have a say in prioritising and developing more appropriate services [48], which could include community-led spaces for knowledge sharing on health and wellbeing. Leveraging existing community networks and capacity, such as engagement with women’s groups and community health champions, could help to raise awareness of screening and motivations to attend screening. Recent systematic reviews have pointed to evidence supporting educational interventions (randomised controlled trials (RCTs) and quasi-RCTs) in small group community settings or with lay health workers; however, the majority were based in the USA or other settings that did not have organised screening programmes [49–51]. Group education and community-led sessions, facilitated by community health workers or ‘community champions’, have been reported elsewhere as an appropriate means to promote screening among low-income groups and align with the co-designed coffee morning’s informal approach [52–54].

Phase 2 participants suggested that to increase acceptability and effectiveness, the coffee morning required a clear pathway for attendees to follow up, book or participate in screening, requiring changes to service provision. As service navigation support is evidenced to increase participation in screening, facilitators of the event should support women to register for screening or suggest sample-takers in their area to book a screening appointment [50]. With the rise in evidence for self-sampling as an effective method of reaching underserved communities [50, 55, 56], there is potential to provide self-sampling kits at community-based educational events [53]. 

### Media campaigns to promote screening

The promotional underwear tag solution is a novel approach to promoting screening. The visual of underwear has been used in several communication and marketing campaigns to promote screening awareness. The NHS recently introduced a new campaign to promote breast screening that associates taking off a bra at the end of the day with the feeling of relief from being screened. The campaign increased traffic to their website by 145% in just the first week [57]. Underwear imagery has also been used for cervical screening campaigns and media advertisements, like the ‘What’s pants, but can save your life?’ campaign in the West Midlands, UK, and a guerrilla campaign called ‘We are all smear ready’ in which women made underwear out of paper and posted them online [58, 59]. Partnerships with retailers are not uncommon for mass media campaigns promoting cancer prevention [60–62]. For example, the UK bowel cancer campaign #GetOnARoll, which involved printing bowel cancer symptoms on toilet roll packaging, has been widely adopted by retailers and increased media attention, but it is unclear the effect it’s had on screening uptake [62]. Acceptability and effectiveness of these campaigns that partner with retailers is unclear, and research is needed to fill this knowledge gap. Other multipronged mass media campaigns for bowel screening in Australia have shown that participation in screening increased during the campaigns, including among those who had never been screened [63, 64].

The limited awareness women had of national cervical cancer prevention and awareness days/weeks and the lack of associated symbols for cervical screening highlight an opportunity to align messaging and promote screening on dedicated days. Breast cancer has a widespread association with the colour pink, whereas cervical cancer’s teal and white ribbon was unfamiliar to the co-designers. The underwear symbol as an association to cervical screening was generally well received but had mixed views on its appropriateness; however, participants struggled to come up with alternative visuals to represent screening. Future work could assess the acceptability of underwear as a symbol for cervical screening or an alternative symbol. Having a visual association, akin to the pink ribbon for breast cancer, may aid the normalisation of cervical cancer public discourse when advertised. However, it is important to ensure campaigns are informative and increase knowledge, rather than more simply persuasive or sales-driven through themed merchandise that do not contribute to the cause [65, 66].

### Strengths and limitations

This study developed two novel co-designed solutions to promote cervical screening, which were developed *by* women of low socio-economic position living in inner-city Dublin. Fundamental co-design principles were upheld through careful consideration of the approach taken to enable equal involvement, manage power dynamics, provide an inclusive environment, and manage expectations [34].

The pragmatist theory-informed approach provides researchers with a template to develop interventions to change behaviours using a co-design approach. Due to its two-phase approach, it outlines a method to be inclusive and considerate of all stakeholder perspectives while enabling the population of interest to have their own space to speak freely about their needs and desires without being constrained by feasibility and difficult power dynamics imposed by other stakeholders. It provides a means to integrate behaviour change theory, acceptability theory (for evaluation) and application of the Double Diamond and PRODUCES + co-design protocol to robustly and transparently conduct co-design research. Specifically, the TFA complements co-design intervention development by outlining a framework to receive feedback from stakeholders and refine the solutions before piloting them. In an ever-expanding landscape with a diverse range of methods that can be applied to co-design, the use of these tools not only increases transparency of the work conducted but also provides useful insights for other researchers working in the field.

Co-design workshops may have benefited from having all relevant stakeholders at the table at the same time; however, when the group were asked if they wanted to invite other stakeholders to the group, they declined as they felt they knew best themselves what was needed in their community. The workshops would have benefited from an additional facilitator whose role was to observe and take notes on the workshop to ensure all perspectives were gathered and incorporated into the outcomes and to evaluate the process. There was no formal evaluation of the co-design process from the perspective of group members, but reflections from the lead researcher and PPI contributors (Appendix 8) saw changes from the first to last workshop, where group members considered themselves to move from sharing perspectives to having ownership over the research, which aligns with reflections from another co-design study [67]. This is evident from a video created on the co-design process highlights the collective ownership and benefits of the co-design approach [68].

Convenience sampling of the PoI participants limited the diversity of the sample and potential acceptability of the solutions to others with intersectional challenges related to place of residence, disability and other factors. The lead researcher and PPI contributors who attended some phase 2 focus groups were challenged not to disclose their opinions or justify reasons for design choices when participants challenged aspects of the solutions, as a sense of ownership of the solutions was developed throughout the co-design workshops. The lead researcher was careful not to overexplain but simply clarify queries about the solutions if participants were unsure of any aspect.

### Implications of the co-designed solutions

Sustained collaboration with existing stakeholders would facilitate knowledge translation and accelerate feedback into practice, and co-design work enables this through sharing power and responsibilities. As implementation and evaluation were beyond the scope of this research, future studies should consider further developing these solutions and testing their effectiveness as they appeared acceptable to a range of stakeholders. The findings from this work identified how the content and delivery of cervical screening communications can be tailored to address the needs of those facing socio-economic disadvantage. Specifically, the coffee morning solution has insights that could enhance a Community Champions initiative that has been piloted in Ireland [53]. As these solutions were developed with a small group of co-designers, further refinement for implementation must engage other marginalised voices, such as those who were never screened, people experiencing homelessness or live with a disability, who may not have been represented in the co-design group and consider intersectional barriers to engagement with screening. Partnership with marginalised voices requires time, resources and willingness to create a safe space to enact change. Without sensitive, clear and engaging communications about screening that are targeted to the specific needs of populations who are underrepresented in screening, it risks diminishing the opportunity for informed decision-making and consent to participate in screening. Involving members of underrepresented communities in the research and design of initiatives to promote cancer knowledge and screening gives agency to the community and democratises health needs, and this approach sets it apart from traditional methods of intervention development [69]. This research highlights the invaluable contribution of local knowledge and provides a template for other researchers to undertake collaborative values-based research.

## Conclusion

This work highlights a novel, theory-informed approach to co-designing health interventions. It provides a template for integrating behaviour theory into co-designed interventions and an approach to gain stakeholder perspectives at various stages of the design process. Two different and creative solutions to promoting cervical screening among those of lower socio-economic position were developed, a coffee morning and a promotional tag for underwear. These solutions highlight the desire for increased visibility to normalise and increase awareness of cervical screening in the public discourse and to create safe spaces for women to discuss health concerns outside of a health setting. These solutions provide recommendations and templates for adaptation to promote screening in other communities and eliminate cervical cancer equitably.

## Supplementary Information

Supplementary Material 1

## Data Availability

All data generated or analysed in the phase 1 study are included in this published article. The phase 2 dataset generated and analysed during the current study are not publicly available due to the potentially identifiable nature of responses due to the limited number of appropriate PPAC participants, but may be available from the corresponding author on reasonable request.

## Footnotes

1 Due to the small sample size of the group and that a video was created in relation to the co-design project, ethnicity was summarised as Irish or non-Irish for confidentiality of the co-designers.
