# Supplementary material for: Co-designing solutions to promote awareness of cervical screening with women of low socio-economic position living in urban areas
Source: BMC Public Health. 2026 Apr 17;26:1742. doi: 10.1186/s12889-026-27144-3 (PMC13220630; doi:10.1186/s12889-026-27144-3)
Supplement: Supplementary file 1 — Supplementary Material 1 [file 12889_2026_27144_MOESM1_ESM.docx]

## Appendix for co-design study

### 1 GRIPP2 checklist

| Section and topic | Item | Reported on section/s |
| --- | --- | --- |
| Section 1: Abstract of paper | |  |
| 1a: Aim | Report the aim of the study | Background |
| 1b: Methods | Describe the methods used by which patients and the public were involved | Methods |
| 1c: Results | Report the impacts and outcomes of PPI in the study | Results |
| 1d:Conclusions | Summarise the main conclusions of the study | Conclusion |
| 1e: Keywords | Include PPI, “patient and public involvement,” or alternative terms as keywords | Co-designers sample |
| Section 2: Background to paper | |  |
| 2a: Definition | Report the definition of PPI used in the study and how it links to comparable studies | NA |
| 2b: Theoretical underpinnings | Report the theoretical rationale and any theoretical influences relating to PPI in the study | Background, Methods |
| 2c: Concepts and theory development | Report any conceptual models or influences used in the study | Background, Methods |
| Section 3: Aims of paper | |  |
| 3: Aim | Report the aim of the study | The challenge |
| Section 4: Methods of paper | |  |
| 4a: Design | Provide a clear description of methods by which patients and the public were involved | Methods |
| 4b: People involved | Provide a description of patients, carers, and the public involved with the PPI activity in the study | Co-designers sample, Phase 2 sample |
| 4c: Stages of involvement | Report on how PPI is used at different stages of the study | Methods |
| 4d: Level or nature of involvement | Report the level or nature of PPI used at various stages of the study | Considerations for the workshops |
| Section 5: Capture or measurement of PPI impact | |  |
| 5a: Qualitative evidence of impact | If applicable, report the methods used to qualitatively explore the impact of PPI in the study | NA |
| 5b: Quantitative evidence of impact | If applicable, report the methods used to quantitatively measure or assess the impact of PPI | NA |
| 5c: Robustness of measure | If applicable, report the rigour of the method used to capture or measure the impact of PPI | NA |
| Section 6: Economic assessment | |  |
| 6: Economic assessment | If applicable, report the method used for an economic assessment of PPI | NA |
| Section 7: Study results | |  |
| 7a: Outcomes of PPI | Report the results of PPI in the study, including both positive and negative outcomes | Results, Strengths and limitations |
| 7b: Impacts of PPI | Report the positive and negative impacts that PPI has had on the research, the individuals involved (including patients and researchers), and wider impacts | Strengths and limitations |
| 7c: Context of PPI | Report the influence of any contextual factors that enabled or hindered the process or impact of PPI | Considerations for the workshops, Strengths and limitations |
| 7d: Process of PPI | Report the influence of any process factors, that enabled or hindered the impact of PPI | Strengths and limitations |
| 7ei: Theory development | Report any conceptual or theoretical development in PPI that have emerged | NA |
| 7eii: Theory development | Report evaluation of theoretical models, if any | NA |
| 7f: Measurement | If applicable, report all aspects of instrument development and testing (eg, validity, reliability, feasibility, acceptability, responsiveness, interpretability, appropriateness, precision) | NA |
| 7g: Economic assessment | Report any information on the costs or benefit of PPI | NA |
| Section 8: Discussion and conclusions | |  |
| 8a: Outcomes | Comment on how PPI influenced the study overall. Describe positive and negative effects | Strengths and limitations, Appendix 15 |
| 8b: Impacts | Comment on the different impacts of PPI identified in this study and how they contribute to new knowledge | Strengths and limitations |
| 8c: Definition | Comment on the definition of PPI used (reported in the Background section) and whether or not you would suggest any changes | NA |
| 8d: Theoretical underpinnings | Comment on any way your study adds to the theoretical development of PPI | Methods, Strengths and limitations |
| 8e: Context | Comment on how context factors influenced PPI in the study | Considerations for the workshops, Strengths and limitations |
| 8f: Process | Comment on how process factors influenced PPI in the study | Considerations for the workshops, Strengths and limitations |
| 8g: Measurement and capture of PPI impact | If applicable, comment on how well PPI impact was evaluated or measured in the study | Strengths and limitations Appendix 6.15 |
| 8h: Economic assessment | If applicable, discuss any aspects of the economic cost or benefit of PPI, particularly any suggestions for future economic modelling. | NA |
| 8i: Reflections/critical perspective | Comment critically on the study, reflecting on the things that went well and those that did not, so that others can learn from this study | Strengths and limitations Appendix 6.15 |

### 2 Facilitator role

- Listen – very active listening to everyone in the group.
- Clear communication/instruction of each activity.
  - Show don’t tell – show people how to do stuff
  - Write out your plan
- Be flexible to change plan.
- Respond to needs of group. If people are tired, suggest a break or move on to a different activity. Just get people
  - A break could be a game that gets people into their body such as keepy uppys with a piece of paper, Body mirroring, Throw a ball
- Keep to time.
- Encourage everyone to speak up – manage by doing individual thinking to group activities. Politely interrupt loud voices and give opportunity to quieter people.
  - Small group discussions and move around to probe quieter voices, then in bigger group you can ask the person to elaborate so more details on topic given
- Create open, safe and inclusive space.
- Encourage diversity of opinion.
- Manage conflicts – reiterate there are no wrong answers, everyone can have their opinion and we can agree to disagree.
- Refer back to ground rules if needed.
- Keep group focused and on track of goals.
- Try to identify consensus and support decisions for progress to next step.
- If someone is uncomfortable or upset, take them aside to see what’s wrong and how they can be supported.
- Probe questions to group. Leave your opinions until after other have spoken or as a way to start conversation if long silence from group.

**Support phone numbers**

General Health:

HSE Live: 1800 700 700

Any GP or primary care centre

Cervical screening available at any GP or women's clinic

Summerhill Primary care centre: 01 707 2300

Women's health:

Well Woman centre, Liffey Street: 01 872 8051

Well Woman, Coolock: 01 848 4511

Irish Family Planning Association, Cathal Brugha Street: 01 872 7088

Irish Family Planning Association, Tallaght: 01 459 7685

Information and concerns about cancer for you or your family:

Irish Cancer Society helpline: 1800 200 700

Cancer diagnosis, treatment, survival support:

ARC support line: 01 215 0250

Mental health support:

Aware: 1800 80 48 48

Samaritans: 116 123

Text about it: Free-text HELLO to 50808

Free Counselling from National Counselling Service with a medical card (need GP referral):

Dublin North and Dublin North City: 01 795 7170

South Dublin and South East Dublin: 01 280 5862

Dublin South West: 01 921 4792

Domestic or sexual Abuse:

Women’s aid: 1800 341 9006.4 Group Contract

### 3 Co-design workshop agendas

#### 3.1 Workshop 1 Agenda

| **Time** | **Activity** | **Lead person, materials** |
| --- | --- | --- |
| **9.30am**  15 mins | Tea and coffee informal chats, registration and consent | Food and drinks laid out, reusable cups for everyone, information sheet and consent form, cervical screening leaflets, battery energy level sheet and evaluation sheet  SMS, RK and NC |
| **9.45am**  10 mins | Intro to project - why are we here | SMS |
| **9.55am**  20 mins | Ice breaker games:  Stop to the music, in pairs answer question  Circle to get to know names, fill out battery energy level | Ball, speaker, timer  SMS, RK and NC |
| **10.15am**  20 mins | Group contract discussion | Flip pad, pens, markers, beanbag/ball  SMS |
|  | Break? |  |
| **10.35am**  45 mins | Presentation of interview findings – reasons for attending or not attending screening  Group discussion of results and visual representations of these reasons via collage | Map of findings  Paper, magazines, images for collage, post-its, pens, markers, paper, scissors, glue, blue tack, tape  SMS, RK and NC |
| **11.20am**  25 mins | Break for sandwiches |  |
| **11.45am**  20 mins | Vote with stickers what’s most important issues  Reflect | Dot stickers, flipchart  SMS |
| Optional/ alternative  15-30 mins | Magic wand to fix a problem with screening | Post-its, flipchart  SMS |
|  | Break? |  |
| **12.05pm**  20 mins | Plan for next session  Evaluation/reflection on day | Flip chart  Evaluation sheet, battery energy level  SMS |
| **12.25pm** | Wrap up and thanks | SMS |
| **12.30pm** | Clean up |  |

#### 3.2 Workshop 2 Agenda

| **Time** | **Activity** | **Lead person, materials** |
| --- | --- | --- |
| **9.30am**  15 mins | Tea and coffee informal chats, registration and consent | Food and drinks laid out, plates, cups, information sheet and consent forms, nametags, cervical screening leaflets, battery energy level sheet  SMS, RK and NC |
| **9.45am**  5 mins | Intro to project, recap on 1^st^ session, group contract | SMS |
| **9.50am**  10 mins | Ice breaker games:  Battery level  Bingo | Ball, speaker, timer, bingo sheets, pens  SMS, RK and NC |
| **10.00am**  10 mins | Reflect on top priorities from 1^st^ session  Intro to touchpoints/problems and change into questions for activity | Poster of results and flipchart with priorities  SMS |
| **10.10 am**  45 mins | How might we…. Questions, 1-2-4-all  (1 mins) Individually:  (2 mins) Share ideas in pairs,  (4 mins) Share ideas in groups of 4 (x2 groups)  (10 mins) Share to whole group and note down ideas on flipchart  X2 | Paper with questions, post-its, markers, pens, stickers,  Music  SMS, RK and NC |
| **10.55 am**  20 mins | Lunch break | Sandwiches, tea, coffee, plates and cups |
| **11.15 am**  40 mins | How might we…. Questions, 1-2-4-all  X2 | Paper with questions, post-its, markers, pens, stickers,  Music  SMS, RK and NC |
|  | Break? |  |
| **11.55 pm**  15 mins | Add anything else to the How could we’s…  Vote on top solutions | Stickers  SMS, RK and NC |
| **12.10pm**  20 mins | Plan for next session  Evaluation/reflection on day  Ask about filming for mini documentary, acceptable? | Flip chart  battery energy level  SMS |
| **12.30pm** | Wrap up and thanks | SMS |
| **12.35pm** | Clean up |  |

#### 3.3 Workshop 3 Agenda

| **Time** | **Activity** | **Lead person, materials** |
| --- | --- | --- |
| **9.30am**  10 mins | Tea and coffee, informal chats, registration | Food and drinks laid out, reusable cups for everyone, registration sheet  SMS, RK and NC |
| **9.40am**  10 mins | Icebreaker/energiser  Battery level  Drawing squiggle bird | Battery level sheets, blank paper, pens  SMS |
| **9.50am**  5 mins | Aim of the day  Recap on previous workshops key problems and solutions | Posters of results from last workshops  SMS |
| **9.55am**  15 mins | Examples of resources to promote screening | Laptop for videos, printed examples and posters |
| **10.10am**  1hr | Develop ideas  First define specific problem and solution  Consider: What, who, where, when, how idea would work - worksheet | Worksheets, paper, pens, markers, collage paper, flipchart  SMS, NC, RK |
| **11.10am**  20 mins | Lunch break  Ask about filming video on project | Teas, coffees, sandwiches |
| **11.30am**  20 mins | Pitch idea to other group  Q&A  Test idea with character profiles | Character profiles sheets  SMS |
| **11.50pm**  30 mins | Reflect on ideas and decide on one idea to move forward with – what, so what, now what worksheets  5 mins alone  10 mins in group  15 Whole group decide on one idea | Reflection sheets  SMS |
| **12.20pm**  10 mins | Wrap up and planning what next:  Another meeting to refine idea, this would be autumn?  Designer to make ideas reality – want to refine them?  Launch event?  Filming  Vouchers  Thanks | SMS |
| **12.30pm** | Clean up |  |

#### 3.4 Workshop 4 Agenda

| **Time** | **Activity** | **Lead person, materials** |
| --- | --- | --- |
| **9.30am**  10 mins | Tea and coffee, informal chats, registration waiver for video | Food and drinks laid out, reusable cups for everyone, registration sheet, Waiver for video  SMS, RK and NC, Hannah Bloom |
| **9.40am**  10 mins | Icebreaker/energiser  Battery level | Battery level sheets, blank paper, pens, speaker  SMS |
| **9.50am**  10 mins | Aim of the day  Recap on previous workshops key problems and solutions and ideas | Posters of results from last workshops  SMS |
| **10 am**  35 mins | Share coffee morning idea and posters  What, so what, now what worksheet | Worksheets, paper, pens, markers, collage paper, flipchart  SMS, NC, RK, Cuan |
| **10.35am**  35 mins | Share underwear tags  What, so what, now what worksheet | Worksheets, paper, pens, markers, collage paper, flipchart  SMS, NC, RK, Cuan |
| **11.10am**  10 mins | What will we do next – what do we ask other stakeholders, do we actually want to do an coffee morning/event? | Flipchart  SMS |
| **11.00am**  20 mins | Lunch break | Food and drinks laid out, reusable cups for everyone SMS, NC, RK |
| **11.20pm**  60 mins | Video interviews | Area set up for interviews and group exercise  Hannah Bloom |
| **12.20pm**  10 mins | Thanks | SMS |
| **12.30pm** | Clean up |  |

### 4 Sample of real-world examples to promote cervical screening


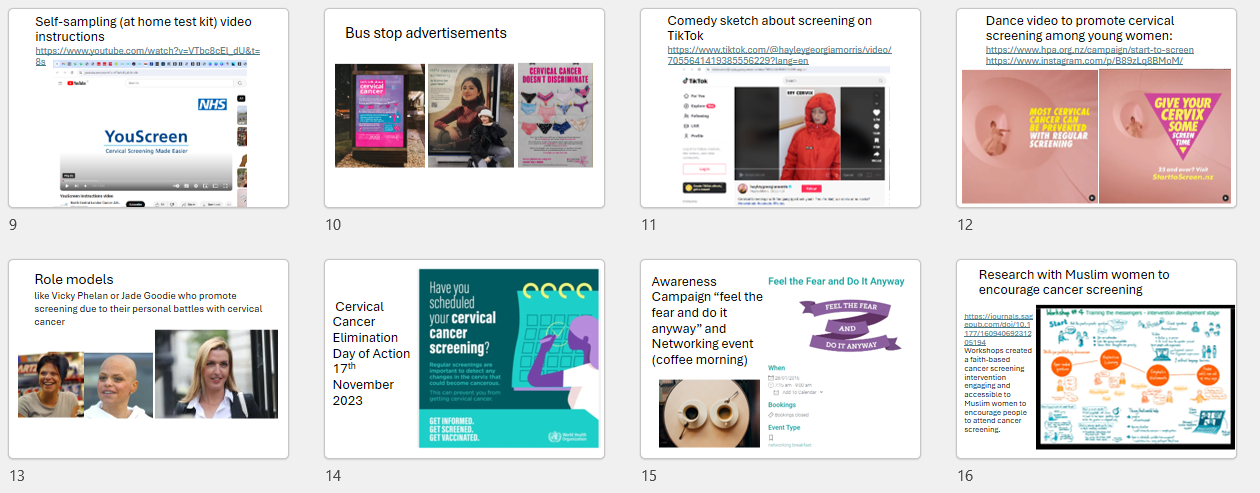


### 5 Worksheet for reflection on solution (Workshop 3)


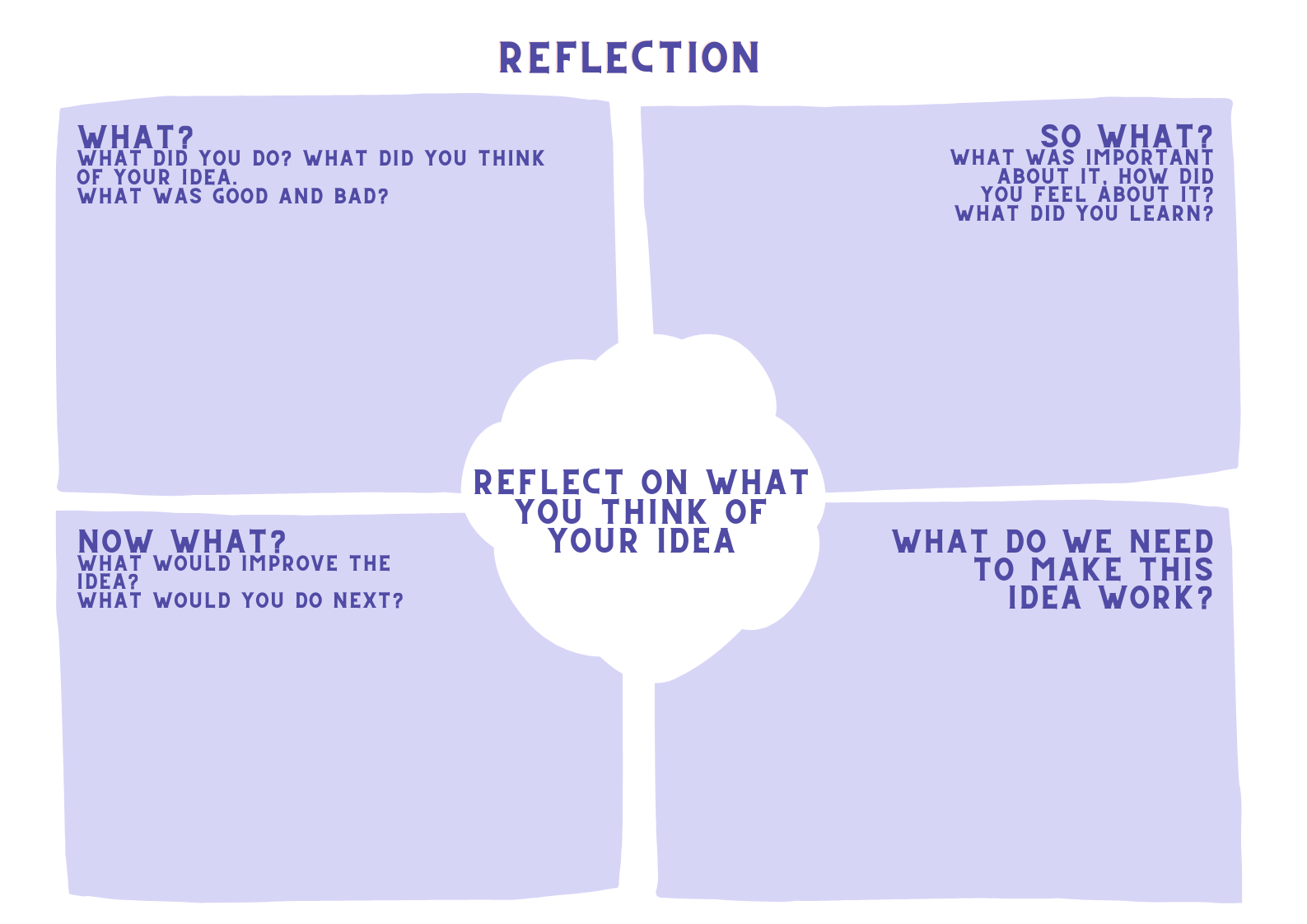


### 6 Worksheet to reflect on mock-up designs (Workshop 4)


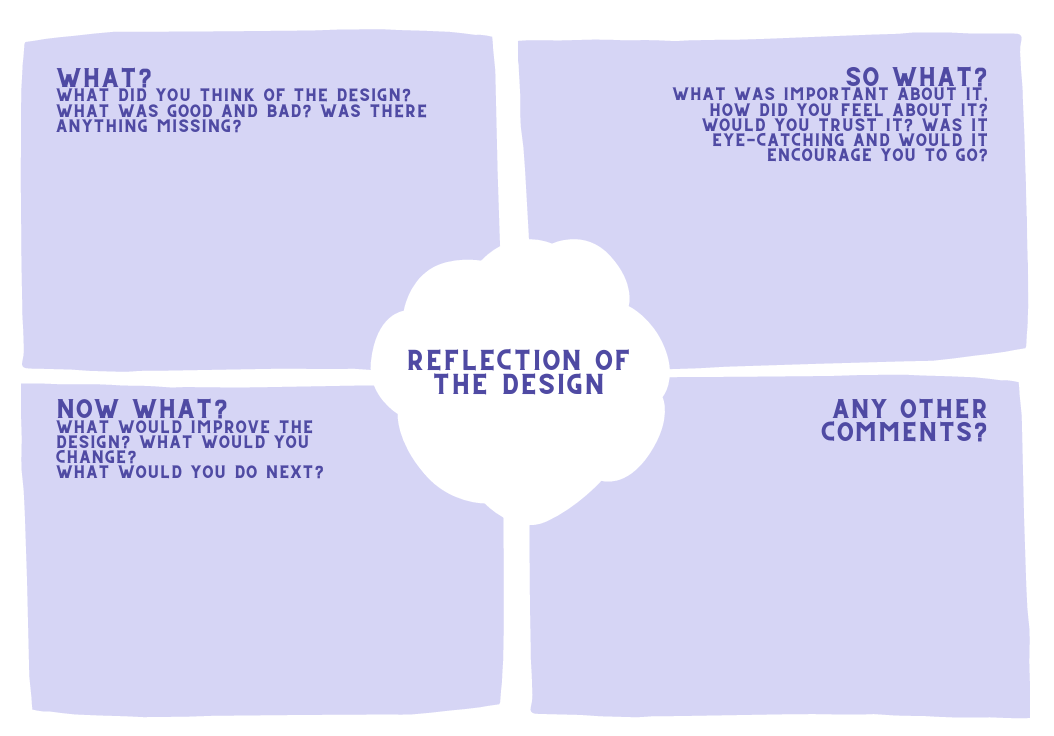


### 7 Focus group and interview topic guide

**Introductions**

Greet the group, introduce yourself (PhD student at UCD, experienced interviewer) and explain the purpose of the focus group is to discuss their views on the two co-designed ideas to improve awareness cervical cancer screening (hypothetical prototypes and still in development). Your names won’t be used, confidential, but answers will be used to improve ideas and be reported. Will explain each idea and then ask for your feedback.

Ask if there are any questions about the research. Check that you have consent. Remind them that this is voluntary, anonymous/confidential, contact details/names held separately to data collected, can withdraw any time and will be recorded.

Explain ground rules of focus group –

- We're on a first name basis
- No right or wrong answers, only your honest opinion
- Respect differing points of view;
- Allow everyone to share their opinion
- You don't need to agree with others, but you must listen respectfully as others share their views;
- As we're tape recording, one person speaking at a time;
- What’s said in the room stays in the room
- Please don’t share out these ideas with others – not public yet
- My role as moderator will be to guide the discussion, talk to each other
- May need to interrupt to stay on topic and get through all answers

Introduce yourselves – names and where you are from

Start recording.

***Questions for stakeholders involved in planning, promotion or delivery***

***(~35mins for each idea)***

***Share co-designed resource with participants***

Describe resource/campaign and pass around mock-up designs

**Views on co-designed resource**

1. What is your overall impression? (attitude, ethicality)
   1. What did you like about it?
   2. What did you dislike about it?
   3. Colour, Font, Style
2. What did you think of the overall message? Was it clear and the information is understandable? (intervention coherence, self-efficacy)
3. Would this encourage women of low SEP to go/promote screening? (effectiveness, self-efficacy)
   1. What makes you say this?
4. Would you encounter any challenges providing it? (Burden, opportunity costs, self-efficacy)
5. What would make this more trustworthy or impactful? (attitude, ethicality)
6. Is there anything that makes you uncomfortable/disagree with/doesn’t sit right on principle? (ethicality)

**Implementation (APEASE practicability, scalability, (cost-)effectiveness)**

1. How do you think this could be put into practice/made a reality? (opportunity costs)
   1. Discuss barriers and enablers.
   2. Any changes or useable as is?
2. How could this be adapted to other audiences?
   1. Do you think it will reach all women (of low SEP) or exclude some people?
   2. How could it be more inclusive? (ethicality)
3. Could this be afforded if delivered at scale?
4. Is it sustainable?
5. Any unintended harms/risks?

**Wrap up**

1. Anything missing you would like to know about or see in this resource/campaign? (attitude)
2. Is there anything you think we missed?
3. Do you have any questions for me?

Thanks very much for speaking with me. Your answers will be used to improve ideas, be reported and given as recommendations. Would you like me to hold onto contact details for the results of this research? Share transcript. If debrief needed concerning worries about cancer, refer to Irish Cancer Society helpline.

***Questions for target population (35 mins for each idea)***

***SMS to share co-designed resource with participants***

Describe resource/campaign and pass around mock-up designs

**Views on co-designed resource**

1. What is your overall impression/opinion? (attitude, ethicality)
   1. What did you like about it?
   2. What did you dislike about it?
   3. Colour, Font, Style
2. Do you think the overall message was clear and the information is understandable? (intervention coherence, self-efficacy)
3. Would this encourage you/your friends and family to go? (effectiveness, self-efficacy)
   1. What makes you say this?
4. Is it easy or difficult to engage with? Would you encounter any challenges taking part/using it? (Burden)
5. Would you trust this if you saw it? (attitude, ethicality)
   1. What would make this more trustworthy or impactful?
   2. Who would you expect to get this from/where would you expect to see/find this information?
6. Is there anything that makes you feel uncomfortable/disagree with on principle? (ethicality)

**Implementation (appease/frame-it)**

1. How do you think this could be done in real life/ in practice? (opportunity costs)
   1. Discuss barriers and enablers.
   2. Do you think it will reach all women or will some be excluded? (ethicality)

**Wrap up**

1. Anything missing you would like to know about or see in this resource/campaign? (attitude)
2. Is there anything you think we missed?
3. Do you have any questions for me?

Thanks very much for speaking with me. Your answers will be used to improve ideas, be reported and given as recommendations. Would you like me to hold onto contact details for the results of this research? Share transcript.

If debrief needed concerning worries about cancer, refer to Irish Cancer Society helpline.

### 8 Reflection on co-design process

#### 8.1 Brief summary of reflections from each workshop

Workshop 1: The group’s decision to not invite other stakeholders was respected, upholding the principles of respecting the group’s opinions and valuing their expertise by experience; that they know best for their community. The group appeared comfortable with each other from the beginning and open to sharing their opinions and personal experiences with the whole group. The group were happy to sit and discuss the results on the mindmap but did not actively engage with the collage or post-it notes to explain their views. The lead researcher, had to adapt to take notes on post-its to add to the mindmap instead. The voting activity with discussion worked well and women were in agreement with the top issues.

Workshop 2: The ‘how might we’ questions may have benefitted from more critical reflection and refinement as there was some overlap in the questions and the group found this challenging to think about the questions differently, potentially asking simply: what should we say and what could we do to promote screening. Women were challenged by the 1-2-4-all activity when working individually. They were reluctant to write down their own ideas but once grouped in pairs and groups the ideas flowed more freely. This technique encouraged the quieter member’s views to be shared.

Workshop 3: The group chose to develop two ideas in the third workshop. This workshop had really positive collaborative engagement and the worksheets worked well to support the group to develop their ideas. However, there was not an opportunity to narrow down to one solution as there was limited time to discuss the pros and cons of each solution during the reflection.

Workshop 4: Having a visual mock-up made the refinement process more tangible for the co-design group as it was easier to talk about what was good/bad about the designs and get to the crux of what they wanted.

#### 8.2 Reflections from PPI contributors

**What did you do for the project (e.g. at the workshops, advisory group meetings and recruitment and advertisement of the project)?**

Reflection 1: I started off with the advisory group on zoom. We all came together to come up with ideas for bringing women together to discuss cervical screening. We then set up the workshops to make the women feel comfortable discussing this topic as it can be quite hard to discuss for some women. It was nice to sit down and discuss what we can do as a group to encourage other women to go for the screening. I found recruiting the people easy as I feel passionate about this topic.

Reflection 2: I took part in about five workshops where I shared my views and experience about cervical cancer with other women. I helped to review materials and give feedback on how they could be made clearer and more understandable to people with low education. I also joined some advisory group meetings online. I listened to updates and how the project was progressing.

**How did you feel about being involved in the project?**

Reflection 1: At first I was really nervous about being involved in the project as I have never done anything like this before. Once I got started I felt part of the group and everyone made me feel very welcome.

Reflection 2: I felt proud and happy to be part of something meaningful and close to my heart. It was rewarding to know I could make a difference for women in and around the area where I live.

**What was good and bad about being involved?**

Reflection 1: I found the information I got from other people in the group very helpful to me, I was able to pass that on to friends and family. Like regarding where women can go for the screening. It was nice being involved with such a great group. I didn't find anything bad about the group but I did find it a little hard at times during the project as I lost my father in law to cancer.

Reflection 2: Good: Meeting with people who had different experiences of cervical cancer screening, learning about HPV and what it is and the difference it can make if caught early, feeling that I have a voice and it was listened to. Bad: I feel there could have been more people involved in the workshops, trying to fit the project in and around my day to day life.

**Is there anything you would have done differently?**

Reflection 1: No, I think it was done very well.

Reflection 2: I don’t think I would as everyone was very comfortable with each other.

**What did you learn from being involved in the project (e.g. about cervical screening, working on research projects, and your skills and interests)?**

Reflection 1: I feel like I have learnt so much about myself from doing the project. I feel my confidence has grown so much and my knowledge of Cervical Cancer is so much better from when I started. Also now I have a better understanding of why women don't talk about it much. As I have sat and listened to them at the workshops. It has been a great experience from start to finish. SMS has been such a great support for us all. I am really thankful to have been part of such a great project. I really hope our ideas of a tag or coffee mornings will get more women to go for screenings and not feel embarrassed.

Reflection 2: I learned how research projects work behind the scenes and how much planning goes into them. The amount of different people that are involved with trying to get women aware of cervical cancer. I learned that I enjoyed being part of group discussions. Through doing this research I have built confidence and communication skills.
